# htrSPRanalysis: An open source R package for expedited analysis of high-throughput binding kinetics data

**DOI:** 10.1371/journal.pcbi.1014581

**Published:** 2026-07-31

**Authors:** Janice M. McCarthy, Kan Li, Georgia D. Tomaras, S. Moses Dennison

**Affiliations:** 1 Department of Biostatistics and Bioinformatics, Duke University, Durham, North Carolina, United States of America; 2 Center for Human Systems Immunology, Duke University, Durham, North Carolina, United States of America; 3 Department of Surgery, Duke University, Durham, North Carolina, United States of America; 4 Duke Human Vaccine Institute, Duke University, Durham, North Carolina, United States of America; 5 Department of Integrative Immunobiology, Duke University, Durham, North Carolina, United States of America; 6 Department of Molecular Genetics and Microbiology, Duke University, Durham, North Carolina, United States of America; University of York, UNITED KINGDOM OF GREAT BRITAIN AND NORTHERN IRELAND

## Abstract

Surface plasmon resonance (SPR) enables label-free detection of binding kinetics and has been widely applied to the biophysical characterization of molecular interactions such as antibody-antigen binding. With the advent of high-throughput SPR (HT-SPR) instruments, hundreds of binding interactions can be detected simultaneously, combining the details of kinetic measurements with the capability of large-panel biomolecule screening. However, binding kinetics analysis for large panels of antibody or antigen often requires a combination of fitting strategies to address different types of sensorgrams. While software packages exist for SPR binding kinetics data analysis, they are associated with a number of limitations: 1) currently most of the software packages are proprietary, prohibiting widespread use; 2) most of the software packages, including open source packages, are designed primarily for low-throughput data analysis, making analyzing a large number of kinetics data sets labor-intensive; 3) the software typically requires multiple iterative user-interface interactions when analyzing large data sets. Here, we present htrSPRanalysis, an open source R package designed primarily for high-throughput binding kinetics data analysis, currently focusing on 1:1 binding analysis. htrSPRanalysis leverages the increasingly commonplace multi-core computing architecture to efficiently analyze a large number of sensorgrams with minimal user-interface interaction. It also offers automated generation of analysis output for all sensorgrams. Furthermore, beyond manual optimization of sensorgram fitting strategies, htrSPRanalysis accelerates the analysis process by providing automated procedures to determine the optimal concentration range, choose the optimal dissociation window for fitting, and detect bulk shift. The high-throughput functionalities and automation of fitting optimization makes htrSPRanalysis especially useful for speeding up data analysis to get results for implementing further steps in therapeutic antibody discovery research.

## Author summary

Surface plasmon resonance (SPR) is widely used to measure the strength and speed of molecular interactions, such as how antibodies recognize their targets. SPR does not require labeling and can provide real-time kinetics information. Recent advances in high-throughput SPR (HT-SPR) instruments allow hundreds of interactions to be measured simultaneously, which is especially valuable for the screening of large panels of biomolecules. Despite these advances, data analysis remains a major challenge. Most existing software is proprietary, designed for small-scale studies, or requires a time-consuming manual effort for analyzing each molecular interaction, limiting the pace and scalability of research. We developed htrSPRanalysis, an open-source R package designed specifically to analyze high-throughput binding kinetics data. The package leverages multi-core computing to analyze data for many molecular interactions in parallel, dramatically reducing processing time. It also includes automated routines for steps to determine the optimal data analysis strategy, including bulk shift correction, optimal concentration range selection, and dissociation window determination, ensuring more consistent and reproducible analyses. By reducing manual effort and providing scalable analysis tools, htrSPRanalysis helps researchers efficiently interpret HT-SPR data, accelerating the discovery and development of therapeutic antibodies.

## 1. Introduction

Detailed understanding of molecular interactions, such as antibody–antigen and drug–target binding, is crucial for developing novel therapeutics. Binding kinetics platforms detect real-time binding and dissociation between molecules. Label-free kinetic platforms enable real-time detection without fluorescence or other labels that might interfere with binding.

Surface Plasmon Resonance (SPR) [1] is a label-free method used to characterize binding kinetics. In a typical experiment, one binding partner (ligand) is immobilized on the SPR sensor chip, and the other partner (analyte) in solution flows over the surface, allowing interaction. The binding follows:


$$\begin{array}{c} A+L\underset{k_{d}}{\overset{k_{a}}{⇌}}A\cdot L \end{array}$$

where *A* is analyte, *L* is ligand, A·L is complex, and $k_{a}$ and $k_{d}$ are the association and dissociation rate constants. When analyte binds ligand, the added mass on the chip surface changes the reflected angle of an incident light beam [2,3]. Within the linear range, the angle change is proportional to mass change and is quantified in resonance units (RU).

To quantify kinetics, data are collected in three phases: 1) baseline, with buffer over the sensor surface, 2) association, with analyte flowed over the surface, and 3) dissociation, with buffer only, allowing bound analyte to dissociate. Titration of analyte over multiple concentrations enables simultaneous analysis of several baseline–association–dissociation cycles, improving estimation of kinetic parameters.

**Fig 1:**
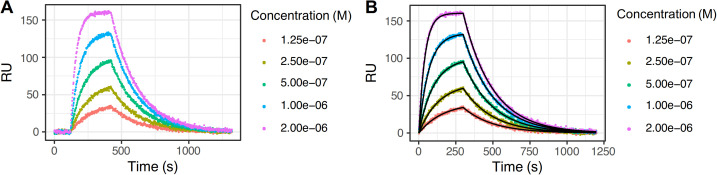
An example SPR kinetics titration sensorgram. Panel A shows the sensorgram before fitting and panel B shows the sensorgram after fitting, with the black curved lines indicating the fitted curves.

A typical titration sensorgram is shown in Fig 1. Usually, a 1:1 binding model is used unless a more complex model is justified. Baselines from each cycle are used for alignment, while association and dissociation data are simultaneously fitted using the Langmuir 1:1 binding model.

Practically, for optimal fitting, several data-selection and fitting details must be considered: 1) For a binding interaction, titrations can span a wide analyte range, but typically only a narrow subset shows strongly concentration-dependent responses suitable for kinetic analysis. This range is usually chosen by visual inspection. 2) Because of factors such as buffer changes, there may be a sudden signal jump between the end of association and the start of dissociation. This gap, termed bulk shift in SPR, should not be fitted as a binding-related signal change. 3) The dissociation phase may plateau at a nonzero response or exhibit multiexponential decay due to nonspecific binding or re-binding, so its length is often truncated by visual inspection for fitting. 4) It can be useful or necessary to titrate multiple analyte concentrations without regenerating the chip surface. In this case, association may not begin at zero RU and must be fitted as a parameter.

High-throughput SPR platforms now enable simultaneous collection of up to 384 titration sensorgrams [4,5], but manual steps in kinetic analysis create a major bottleneck. To better automate fitting for high-throughput data, useful fitting practices must be incorporated.

Current software [6–8] is proprietary, restricted to low-throughput data, or both (Table 1). To our knowledge, the only open-source tools for fitting sensorgrams are Anabel [6], provided as an R Shiny [9] web app and for local use, and a newer web-based Python application [8]. Although these tools have user-friendly interfaces, they lack high-throughput capabilities such as algorithmic selection of the optimal concentration window, automatic bulk-shift detection, and detection of the end of dissociation. None of the existing packages support simultaneous fitting of multiple sensorgrams using the multiple CPUs available on most modern computing platforms.

**Table 1:** Summary of available software for binding kinetics titration analysis. “Multi-core” support refers the software’s ability to leverage multiple CPUs on a computer or on high-performance computing clusters to analyze multiple sensorgrams simultaneously, speeding up computation as the number of available CPUs increases, although the speed-up is sub-linear.

Software	Price	Support for non-regenerative cycles	Fit multiple spots in one run	Multi-core processor support	Platforms	Open source
htrSPRanalysis	Free	Yes	Yes	Yes	All	Yes
TitrationAnalysis [10]	Free	Yes	Yes	No	All	No
Anabel [6]	Free	Yes (Single Cycle Kinetics)	No	No	All	Yes
Web-based app [8]	Free	No	No	No	All	Yes
BIAEvaluation [7]	Supplied by device vendor	Yes (Single Cycle Kinetics)	No	No	Windows	No
Kinetics [11]	Supplied by device vendor with Carterra LSA	Yes (only for 1:1 fit)	Yes	No	Windows	No
Octet [12]	Supplied by device vendor	No	Yes	No	Windows	No
TraceDrawer [13]	$470/yr academic license	No	No	No	Windows	No
Scrubber2 [14]	$490/yr academic license	No	No	No	Windows	No

Previously, we reported a Wolfram Mathematica–based package, *TitrationAnalysis*, that automated high-throughput kinetic analysis, including SPR titration data [10]. The package produced accurate kinetic estimates while reducing repeated manual interaction with software for large data sets. However, Wolfram Mathematica is not freely available, restricting broader use of *TitrationAnalysis*.

Here, we introduce an R-based package for SPR binding kinetics analysis that provides the features above, which are lacking in other software. We further improved on *TitrationAnalysis* capabilities and implemented additional analysis functions in R, a freely available language. In this first version of htrSPRanalysis, we implement only the Langmuir 1:1 [15–18] binding model. The 1:1 model can analyze most kinetic data unless a more complex model can be explicitly assumed.

## 2. Design and implementation

### 2.1. Package overview

The package currently implements the Langmuir 1:1 model to analyze all sensorgrams. The package uses the following equations to fit the association and dissociation phases of the sensorgrams. The association phase was generated from the following equation:


$$\begin{array}{c} RU_{i}(t)=\frac{R_{max}^{i}k_{a}[A{]}_{i}}{\left(k_{a}[A{]}_{i}+k_{d}\right)}\left(1-e^{-(k_{a}[A{]}_{i}+k_{d})(t+t_{0}^{i})}\right)+R_{bulk}^{i} \end{array}$$

where $k_{a}$ is the association rate constant and $k_{d}$ is the dissociation rate constant. $[A{]}_{i}$ is the molar concentration of analyte for a given titration cycle and $R_{max}^{i}$ is the maximum achievable response for the same titration cycle. If we assume the same $R_{max}$ for all analyte concentrations, the term $R_{max}^{i}$ becomes $R_{max}$. If the titration is nonregenerative, $t_{0}^{i}$ fits for a hypothetical time point when the response can be extrapolated to zero. If the titration is regenerative, $t_{0}^{i}$ is zero for all analyte concentrations. If we assume bulk shift occurred due to factors such as buffer mismatch, $R_{bulk}^{i}$ is the bulk shift for the current analyte cycle. If we assume no bulk shift, $R_{bulk}^{i}$ becomes zero for all analyte concentrations.

The dissociation phase is modeled by the equation below, which results in an exponential decay model.


$$\begin{array}{c} RU_{i}(t)=\frac{R_{max}^{i}k_{a}[A{]}_{i}}{\left(k_{a}[A{]}_{i}+k_{d}\right)}\left(1-e^{-(k_{a}[A{]}_{i}+k_{d})(t_{asso}+t_{0}^{i})}\right)e^{-k_{d}(t-t_{asso})}+R_{drift}^{i} \end{array}$$

where $t_{asso}$ is the time at the end of the association phase. $R_{drift}^{i}$ accounts for any abrupt drift in signal between association and dissociation due to factors such as nonspecific binding during association. In practice, simultaneous fitting for $R_{bulk}^{i}$ and $R_{drift}^{i}$ leads to over-parametrization. Therefore, only one of the two terms can be fitted in a single sensorgram.

The package requires the user to provide an Excel spreadsheet containing the underlying time series for all sensorgrams as well as an Excel spreadsheet containing the fitting instructions for each of the sensorgrams. Currently, the package is designed particularly with the Carterra LSA platform in mind but can be adapted for other platforms. For example, data from Octet Biolayer Interferometry (BLI) instruments (see S1 Data) or from Biacore SPR instruments can be reformatted to use in htrSPRanalysis. Specifically for Carterra LSA, the package determines the region of interest (ROI), *i.e.*, spots on the chip surface corresponding to each time series using the header information from the Carterra output. The user needs to provide information during the immobilization setup (block and 96-well plate position) for the package to match the time series of a given ROI with the correct sample information. To analyze data from a different platform, the user is expected to indicate an assumed ROI for each sensorgram when providing fitting instructions. Each sensorgram can only be fitted once per fitting session.

**Fig 2:**
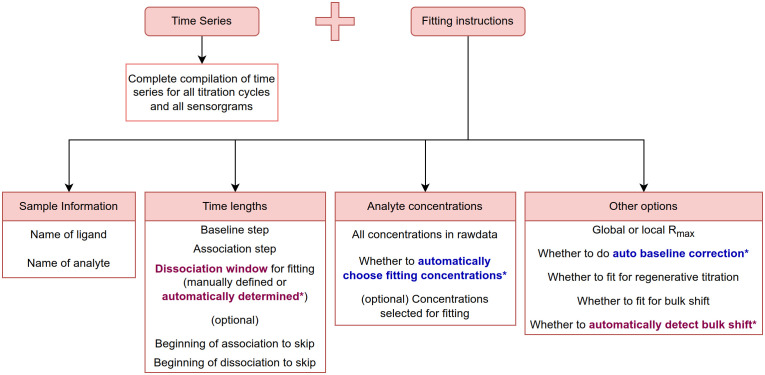
A flowchart of required information from users to use the htrSPRanalysis package. Bold text in blue followed by asterisks indicate automation features adopted from *TitrationAnalysis* into htrSPRanalysis. Bold texts in purple followed by asterisks indicate automation features uniquely developed in the htrSPRanalysis package.

File formatting details and sample files are available when installing the package (see Supplementary Material). Briefly, the fitting instructions for each sensorgram should include (Fig 2): ligand and analyte names; time lengths of baseline, association, and dissociation steps; (optional) time to skip at the start of association and dissociation to avoid artifacts; all analyte concentrations in the raw data; up to five concentrations to be fitted; whether to fit for bulk shift, regenerative titration, automatic baseline correction, global $R_{max}$, automatic bulk shift detection, and automatic dissociation window selection. The dissociation time can be a user-specified value or the full data length if automatic dissociation window selection is activated. If no specific analyte concentrations are chosen, the algorithm automatically selects five consecutive optimal concentrations or uses all concentrations if fewer than five were titrated.

**Fig 3:**
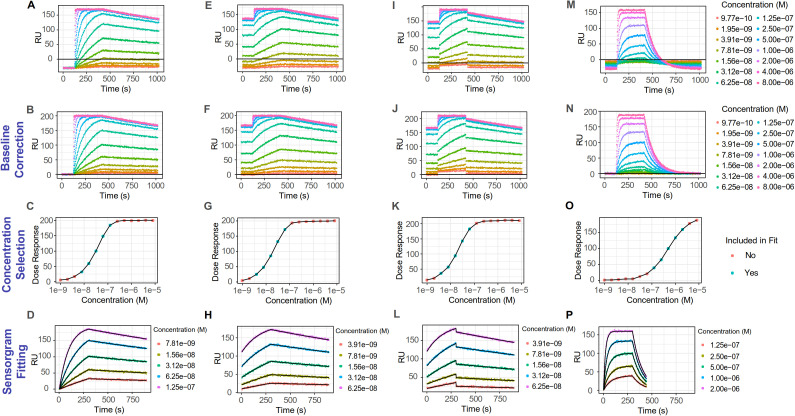
Illustration of the analysis pipeline using simulated data. Each column shows all the analysis steps of a single sensorgram and each row shows a single analysis step for all sensorgrams. All 4 sets of data were simulated using $R_{max}=200$. Data shown in Panel A-L were simulated with $k_{a}=8\times 10^{4}M^{-1}s^{-1}$ and $k_{d}=3\times 10^{-4}s^{-1}$; data shown in panel M-P were simulated with $k_{a}=2\times 10^{4}M^{-1}s^{-1}$ and $k_{d}=1\times 10^{-2}s^{-1}$. In panel C, G, K and O, points in green indicate concentrations selected for fitting. All panels in the figure were generated using the htrSPRanalysis package.

We outline the analysis pipeline and describe key parts in detail in the following subsections. A typical analysis pipeline is illustrated in Fig 3. After the user provides the raw data file and the fitting instructions file, the input files are screened for valid formatting and then processed in R for analysis.

Several analysis steps will be automatically performed for each set of sensorgrams. First, a baseline correction is made for each spot using all the provided analyte concentrations. Second, the 5 “best” concentrations are chosen for curve fitting. In a titration where a two-fold dilution series is used for the analyte, this 5-concentration range is usually equal to or similar to the linear range of the dose response ($\log_{10}$ analyte concentration versus end of association response). After the first two steps, based on the user’s description of the sensorgram, the selected sensorgram is then fitted to a 1:1 Langmuir binding model [15–18] using the nls.lm function from the R MINPACK library [19,20] This function implements the Levenberg-Marquardt optimization algorithm [21,22] for nonlinear least squares curve fitting. If the user specifies in the sample information that the software should automatically determine the optimal dissociation window or detect bulk shift, the corresponding algorithms will be applied to adjust the fitting strategy.

After fitting all sensorgrams, a csv file is produced that contains estimates for $k_{a}$, $k_{d}$, $R_{max}$ (and the bulk shift, if included) and standard errors for all parameters. A PDF file is also produced that outputs one page per sensorgram, including the fitted sensorgram, a table of parameter estimates with standard error, the dose response curve and a plot of fitted residuals.

Some R objects will be stored throughout the analysis pipeline, including:

1. **processed_input**: raw time series and fitting instructions stored in R after format checking and re-organizing using function process_input();
2. **plots_before_processing**: plots of raw sensorgrams before any processing, including baseline correction, obtained by executing function get_plots_before_baseline():;
3. **fits_list**: parameter estimates for each sensorgram obtained by executing the function get_fits();
4. **plot_list**: plots for fitted sensorgrams, obtained using the function get_fitted_plots();
5. **rc_list**: plots for dose-response curves (analyte concentration vs. peak response), with concentrations used in fitting indicated, obtained using the function get_rc_plots()

The user can also create the aforementioned CSV and PDF report using functions create_csv() and create_pdf().

#### 2.1.1. Baseline correction.

To use automatic baseline correction, the user should provide the starting time and time length for baseline averaging. The baselines are averaged for each concentration, and the correction is performed as follows: If the chip surface was regenerated before the start of each cycle, all cycles are adjusted to an average baseline of zero (Fig 3A,3B). If the chip was not regenerated, the software checks to see if the highest baseline has an average that is negative, which most likely indicates that the binding was too weak for analyte to accumulate on the surface after the dissociation step. In this case all cycles are also adjusted to an average baseline of zero (Fig 3M,3N). Otherwise, the baseline average of the lowest baseline among all the cycles is subtracted from the responses (Fig 3E,3F,3I,3J).

#### 2.1.2. Concentration selection.

The user may opt to choose the concentrations to fit by indicating those in the sample information. If not, the package will determine the concentration range in the following manner if data for more than 5 analyte concentrations are provided: The peak binding responses (averaged responses from the last 15 seconds to the last 5 seconds of the association phase) are calculated for each concentration. The consecutive differences between these average responses are recorded, and then a cumulative sum is performed over the differences for each 5 cycle window. The selected concentration range is then set to the 5 cycle window that results in the largest cumulative sum of differences (Fig 3C,3G,3K,3O). This concentration range should be the same as or close to the optimal concentration range. The user can further manually remove concentrations or choose a different range for fitting.

#### 2.1.3. Bulk shift.

Bulk shift usually occurs when there is a buffer mismatch between the association step and baseline/dissociation step, causing a sudden change in signal independent from signal change due to binding or dissociation. The user can choose to have bulk shift fitted or have the algorithm determine whether bulk shift fitting is needed.

Automatic detection of bulk shift is performed per titration cycle in the following manner: First, we compute the average response over the last 50 seconds of the association phase and the first 50 seconds of dissociation. If, for any cycle, the absolute value of the difference between the two averages is greater than a user-defined fold change (1.1 fold by default as a conservative starting value) compared to the standard deviation of the last 50 seconds of association or the first 50 seconds of dissociation, the bulk shift will be included in the fit (Fig 3L). Thus, the determination is attenuated to the noise in the data. The time length used to calculate standard deviations is also tunable by users.

#### 2.1.4. Dissociation window.

There are instances where the user might not want to include the full dissociation window for curve fitting. For example, when there is a very fast dissociation, a large part of the dissociation phase will be flat, and the only variation is due to noise. Another reason to shorten the dissociation window is when there is nonspecific binding causing an upward drift of signal, or there is a bivalent effect (for example, when the analyte is an IgG molecule) and there is an apparent two-phase dissociation.

The user may specify whether the software should determine an optimal dissociation length for curve fitting or to use a provided value. For automatic determination, the software performs a rolling linear regression on the dissociation phase data with a window size of 20 consecutive data points. We begin at the first observed value in the dissociation phase and do linear regression for the first 20 data points. We then begin with the second observed value in the dissociation phase and do linear regression for the second time points to the $21^{st}$, etc. The maximum absolute value of the resulting slopes is computed and used to truncate the dissociation window for fitting when the slope is less than a user-specified fraction of the maximum (30% by default to ensure the rapidly-changing region of the curve is retained) (Fig 3P). This algorithm can be used to exclude later portion of dissociation that is more susceptible to binding artifacts such as baseline drift and detect departures from simple exponential decay such as biphasic dissociation.

The package also detects and truncates upward drift: from the same rolling-slope series, it identifies the consecutive positive slopes and truncates the dissociation window at the start of the analysis, recording an updrift flag for the affected sensorgram. The package has also set the slowest dissociation rate threshold ($1\times 10^{-5}$ $s^{-1}$ as default) to eliminate biophysically unrealistic dissociation rate due to noise or upward drift. The slope-flattening fraction for no-upward-drift dissociation curves (dissociation_slope_tolerance, default 0.30), the number of consecutive positive-slope windows used to declare updrift (upward_slope_window, default 5), and the response-range thresholds for applying automatic window detection (min_RU_tol, max_RU_tol) can all be adjusted by the user in process_input.

#### 2.1.5. Global and local $R_{max}$.

The package provides the option to estimate the $R_{max}$ value for each cycle independently (local $R_{max}$) or estimate a single $R_{max}$ that works for all cycles (global $R_{max}$). In some cases, the 1:1 binding model can be used as a simplified model to quickly fit kinetics traces that contain more complex binding dynamics, where the association phase cannot be adequately explained using a global $R_{max}$ and a local $R_{max}$ estimate for each cycle separately might provide better curve fitting.

### 2.2. Multi-core processor support

In addition to automation features, the package allows for parallelization to take advantage of multi-core systems. Most modern computers are multi-core, meaning they have more than one CPU. High-performance compute clusters typically have 16 + cores, with some as high as 100 cores. By default, the number of available cores is automatically detected when using the package, and all CPU-intensive functions are parallelized to that number of cores, so that many sensorgrams can be analyzed simultaneously. For example, if 32 cores are available, 32 sensorgrams can be analyzed at the same time. The resulting reduction in total run time is sub-linear to the number of cores due to computation needed beyond the fitting step. Users can override the default by setting the “ncores” input within the process_input function.

## 3. Results

Here, we have applied the htrSPRanalysis package to analyze select antibody-antigen binding data sets previously analyzed for the COVIC consortium (covic.lji.org) [4], where we had determined binding affinities and specificities for a large panel (400) of SARS-CoV-2 specific antibodies. These monoclonal antibodies (mAbs) were chosen solely to highlight the performance of the htrSPRanalysis package, and not to indicate that these mAbs are unique compared to similar mAbs in the same study or they are selected for further development.

**Fig 4:**
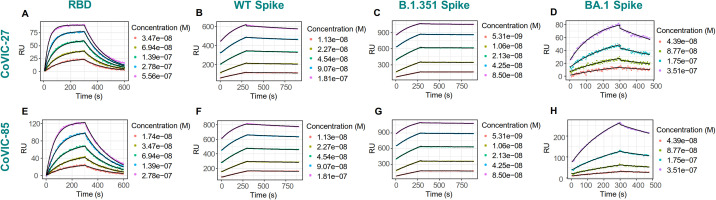
Representative fitted sensorgrams of 2 RBD cryptic site specific mAbs binding to RBD and multiple spike variants. A-D) The sensorgrams of CoVIC-27 binding to RBD, WT spike protein, B.1.351 spike protein and BA.1 spike protein, respectively, are shown. E-H) The sensorgrams of CoVIC-85 binding to RBD, WT spike protein, B.1.351 spike protein and BA.1 spike protein, respectively, are shown.).

To illustrate the general fit performance of the package, we chose to highlight the data from two mAbs as examples. The spike protein is the surface protein of the SARS-CoV-2 virus that caused a COVID-19 pandemic [23,24]. The cryptic binding site on the receptor binding domain (RBD) is a relatively conserved region among the different SARS-CoV-2 variants [25–27] and mAbs that target this site are very likely to bind with strong affinities to multiple spike variants. The representative sensorgrams and triplicate averaged kinetics estimates of two cryptic-site targeting mAbs binding to RBD and multiple spike variants are shown in Fig 4 and Table 2.

**Table 2:** Summary of triplicate averaged kinetics estimates of 2 RBD cryptic site specific mAbs binding to RBD and multiple spike variants. Column titles indicate the names of the mAbs and row titles denote the names of antigens.

Antigen	CoVIC-27			CoVIC-85		
	$k_{a}(M^{-1}s^{-1})$	$k_{d}(s^{-1})$	$K_{D}(M)$	$k_{a}(M^{-1}s^{-1})$	$k_{d}(s^{-1})$	$K_{D}(M)$
**RBD**	$4.59\times 10^{4}$	$6.76\times 10^{-3}$	$1.48\times 10^{-7}$	$3.58\times 10^{4}$	$5.42\times 10^{-3}$	$1.51\times 10^{-7}$
**WT Spike**	$2.43\times 10^{4}$	$8.73\times 10^{-5}$	$3.59\times 10^{-9}$	$2.67\times 10^{4}$	$6.92\times 10^{-5}$	$2.59\times 10^{-9}$
**B.1.351 Spike**	$6.04\times 10^{4}$	$1.58\times 10^{-5}$	$2.62\times 10^{-10}$	$6.02\times 10^{4}$	$1.63\times 10^{-5}$	$2.71\times 10^{-10}$
**BA.1 Spike**	$1.55\times 10^{4}$	$1.37\times 10^{-3}$	$8.96\times 10^{-8}$	$8.12\times 10^{3}$	$1.02\times 10^{-3}$	$1.26\times 10^{-7}$

The kinetics estimates obtained using the htrSPRanalysis package matched previously reported values for these binding interactions (covic.lji.org) [4], which were analyzed using *TitrationAnalysis* [10]. Kinetic estimates show that the two mAbs, CoVIC-27 and CoVIC-85, are capable of retaining strong binding affinities (< 1 nM) to the B.1.351 (Beta) variant and decent binding affinities (89–126 nM) to the BA.1 (Omicron) variant.

Because automatic bulk shift detection and automatic dissociation window determination are unique features implemented in htrSPRanalysis, we will discuss the performance of these features below using other antibody-antigen binding data from the COVIC consortium project.

### 3.1. Automatic bulk shift detection

**Fig 5:**
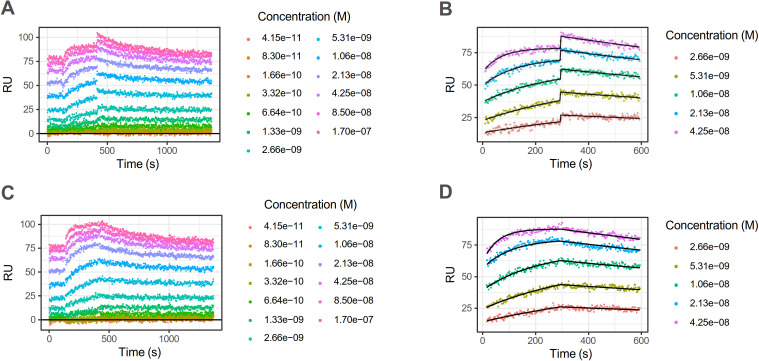
Representative sensorgrams with bulk shift. The figure shows the analysis of binding kinetics between CoVIC-53 and B.1.351 spike protein. Panel A-B show the analysis of binding kinetics with automatic bulk shift detection. Panel C-D show the analysis after the end of association was manually aligned with the beginning of dissociation (inter-step correction).

Fig 5 shows an example sensorgram with bulk shift between association and dissociation (Fig 5A-5B). The htrSPRanalysis package was able to automatically detect and fit for bulk shift. The resulting kinetics estimates were comparable to the kinetics estimates obtained after the bulk shift was artificially corrected (Fig 5C-5D, Table 3).

**Table 3:** Summary of kinetics estimates of example binding sensorgrams with bulk shift.

	$k_{a}\;({𝐌}^{-1}\;{𝐬}^{-1})$	$k_{d}\;({𝐬}^{-1})$	$K_{D}\;(𝐌)$
**Auto Bulk Shift**	$3.35\times 10^{5}$	$3.33\times 10^{-4}$	$9.95\times 10^{-10}$
**Bulk Shift Removed**	$5.25\times 10^{5}$	$3.28\times 10^{-4}$	$6.25\times 10^{-10}$

### 3.2. Automatic dissociation window selection

**Fig 6:**
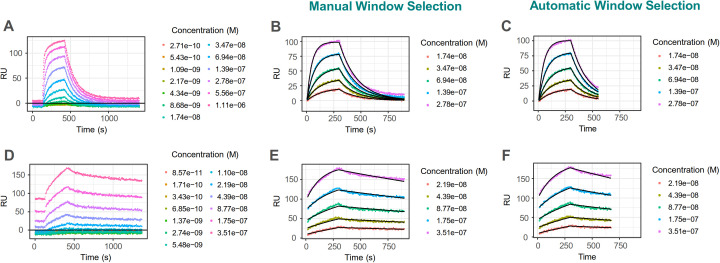
Representative sensorgrams with complex dissociation curvatures. Panel A-C show the analysis of binding kinetics between CoVIC-75 and RBD. Panel D-F show the analysis of binding kinetics between CoVIC-47 and BA.1 spike protein.

Fig 6 and Table 4 show two examples where the dissociation curve cannot be adequately fitted with a single exponential component in its entirety. In the case of CoVIC-75 binding to RBD (Fig 6A-6C), some residual amount of analyte was left bound to the ligand at the end of dissociation. In the case of CoVIC-47 binding to BA.1 spike protein (Fig 6D-6F), the dissociation contained two different curvatures, indicating biphasic dissociation. If 600 seconds of dissociation window was fitted, (Fig 6B,6E), the fittings of dissociation were not ideal for both sensorgrams. The automatic window selection option in the htrSPRanalysis package was able to select windows that were dominated by the first dissociation curvature (Fig 6C,6F)using the default setting. In the case of CoVIC-47 binding to BA.1 spike protein (Fig 6D-6F), the window truncation also improved the fit of the association phase.

**Table 4:** Summary of kinetics estimates of example binding sensorgrams with complex dissociation profile.

mAb-Antigen pair	Fitted using 600 second window			Fitted after auto dissociation window selection		
	$k_{a}(M^{-1}s^{-1})$	$k_{d}(s^{-1})$	$K_{D}(M)$	$k_{a}(M^{-1}s^{-1})$	$k_{d}(s^{-1})$	$K_{D}(M)$
**CoVIC-75 - RBD**	$5.76\times 10^{4}$	$5.98\times 10^{-3}$	$1.04\times 10^{-7}$	$5.00\times 10^{4}$	$6.62\times 10^{-3}$	$1.32\times 10^{-7}$
**CoVIC-47 - BA.1**	$2.07\times 10^{4}$	$3.13\times 10^{-4}$	$1.51\times 10^{-8}$	$1.72\times 10^{4}$	$4.38\times 10^{-4}$	$2.55\times 10^{-8}$

### 3.3. Multi-core performance

To illustrate the speed-up afforded by parallelization, we analyzed subsets of a 384-spot Carterra LSA data set ranging from 64 to 384 sensorgrams, repeating the complete analysis pipeline across a range of core counts on a single compute node. These runs were performed on a high-performance compute node running linux. Table 5 reports the resulting total wall-clock times. Increasing the number of cores substantially reduces run time, but the gains are sub-linear and diminish beyond roughly 16–20 cores, reflecting the fixed serial portions of the pipeline such as input processing and report generation.

**Table 5:** Total wall-clock time (seconds) for the complete htrSPRanalysis pipeline as a function of the number of sensorgrams analyzed (rows) and the number of processor cores used (columns). Values are averaged over replicate runs on a single compute node, each analysis run in a fresh R session.

Number of Sensorgrams	4 cores	8 cores	16 cores	20 cores	32 cores	40 cores
64	126	99	92	81	91	96
96	177	133	116	107	125	111
128	216	161	137	130	142	140
192	317	233	189	180	200	175
256	422	304	235	221	221	253
320	527	365	299	276	274	269
384	635	447	347	337	320	323

Note that analyzing 384 sensorgrams using 40 cores took 323 seconds (5.4 minutes). Comparatively, analyzing the same amount of sensorgrams using the Mathematica package *TitrationAnalysis* on a single core took 30 minutes at a minimum.

## 4. Discussion

The SPR binding kinetics assays can be used to measure a wide range of binding affinities and diverse binding kinetics. Therefore, different fitting strategies must be applied to accurately estimate the kinetics rate constants. Here, the htrSPRanalysis package provides a useful tool for high-throughput analysis of binding kinetics data.

The package provides functions to automatically select concentration range, truncate dissociation window and detect bulk shift. In particular, automatic detection of dissociation phases and bulk shift are uniquely offered in this package. These automatic functions can be used to minimize the user’s need for visual inspection of each sensorgram before fitting and provide guidance for the best fitting strategies. We did find that not all sensorgrams containing bulk shift can be detected automatically and the bulk shift detection algorithm will be further optimized in the future.

The package also provides options to store fitting details for quick recall. While users may follow a simple pipeline outlined in the R vignette (provided in the Supplementary Materials and in the installed R package), there is also tremendous flexibility for more experienced R users. The R objects returned from the main user functions all contain list objects that can be accessed by the user. In particular, the plot functions return a list of ggplot2 [28] objects that can be modified by adding additional layers. Additionally, raw input is accessible in the R object processed_input, and fit results are accessible in the R object fits_list.

Although the package is currently designed with one specific experimental platform in mind, the user can potentially reformat data from other instruments to use with the package.

## 5. Availability and future directions

We have developed a freely available open source R package for the analysis of high-throughput SPR data. The software not only expedites analysis by allowing the use of multiple processor cores to speed up curve fitting, but is also specifically designed to automate conventionally manual steps in the analysis pipeline.

The software is available on the Comprehensive R Archive Network (CRAN) and may be installed using the R command install.packages(“htrSPRanalysis”).

In the current version of the htrSPRanalysis, we have focused on the implementation of 1:1 binding model. More complex binding models may be incorporated into the package in the future. For example, we have previously developed a separate bivalent analyte model [29]. We plan to further optimize the performance of the bivalent analyte model fitting in terms of speed, make it more appropriate for high-throughput SPR and integrate it in a future version of this package. We also plan to incorporate mass transport model into the package in the future after ensuring algorithm stability and parameter identifiability, especially for mass transfer coefficient.

## Supporting information

S1 Text R vignette for htrSPRanalysis. (DOCX)

S1 Data An example of adapting BLI data for use in htrSPRanalysis. (XLSX)

S1 Code htrSPRanalysis code (TAR)
