# Supplementary material for: htrSPRanalysis: An open source R package for expedited analysis of high-throughput binding kinetics data
Source: PLoS Comput Biol. 2026 Jul 31;22(7):e1014581. doi: 10.1371/journal.pcbi.1014581 (PMC13446736; doi:10.1371/journal.pcbi.1014581)
Supplement: Supplementary file 2 [file pcbi.1014581.s003.tar › htrSPRanalysis/inst/doc/htrSPRanalysis-vignette.html]

Introduction to htrSPRanalysis


# Introduction to htrSPRanalysis

This R package consists of a library of functions for processing and analyzing data from SPR binding kinetics experiments, and also contains an example script that executes those functions in a basic pipeline.

Install using install.packages(htrSPRanalysis)

Then load the library using:

```
library(htrSPRanalysis)
```

## Input

The user must provide two input files

1. A time series file that contains the times and corresponding response unit (RU) values for a complete high-throughput SPR run. The software assumes this file is in the output format from the Carterra LSA, with 384 spots on the chip. There are two header lines, as shown below:

2. A sample information file in the form of an Excel spreadsheet with the following columns

### Sample information column definitions

- Incl. - Whether or not to include this sample
- Block/Chip/Tray - The block the sample is in. This may be any combination of 1,2,3,4 - separated by commas. Use multiple values for replicates
- Position/Channel/Sensor - The sample well id in the format of a letter (A-H) followed by a number (1-12). The user may enter ranges (for example A1-B12) when there are replicates. The software will automatically generate a row in the sample information data frame for each of the 384 spots on the chip.
- Analyte - the name of the analyte
- Ligand - the name of the ligand
- Baseline - the length of the baseline data collection phase
- Association - the length of the association phase
- Dissociation - the length of the dissociation phase
- Assoc. Skip - the length of time to skip in the beginning of the association phase. Not currently implemented
- Dissoc. Skip - the length of time to skip in the beginning of the dissociation phase. Not currently implemented
- All Concentrations - the concentrations of analyte used. This is a list, separated by commas. It *must* be the same length as the number of time series corresponding to this spot.
- Incl. Concentrations - either a comma separated list of 4 or 5 consecutive concentrations to use for curve fitting *or* the string “All”. If “All” is entered, the software will automatically choose 4 or 5 optimal concentrations, as described in the documentation.
- Bulkshift - whether or not to force a fit of bulk shift. Value should be “Y” or “N”
- Regen. - whether or not the surface of the chip is regenerated between cycles
- Bsl Start - start time for using the baseline data to perform baseline correction, if the entire baseline is not to be used
- Base Corr - whether or not to perform baseline correction
- Global Rmax - if set to “Y”, the Rmax will be fit globally (one Rmax for all concentrations), otherwise Rmax is fit locally (one per concentration)
- Automate Dissoc. Window - whether or not to automate the length of dissociation phase data used in curve fitting.
- Automate Bulkshift - whether or not to automatically detect bulk shift

### Reading and processing input files, obtaining user supplied options

The function `process_input` takes the paths to the sample information file and the titration data file as its arguments (`sample_sheet_path` and `data_file_path`). It also accepts settings such as the minimum and maximum RU to use for the dissociation window determination, the maximum iterations and the tolerances `ptol` and `ftol` to use for the non-linear least squares curve fit. All of these options are function arguments with sensible defaults, documented in the package help (`?process_input`) and available via tab completion in RStudio. The function returns a `list` object with the sample information, the original time series, the baseline adjusted RUs and several other variables used internally. This is the object that will be passed to the functions for fitting and plotting.

As soon as these files are selected, the software will read in both files and check that the formats are correct. This may take some time, depending on the size of the titration data file.

The minimum allowed \(k\_d\), set with the `min_allowed_kd` argument, is used in the curve/parameter fitting as a lower bound.

The maximum number of iterations (`max_iterations`) is the number of iterations at which the optimization algorithm should stop if convergence is not obtained.

The automatic detection of the dissociation window will be suppressed if the maximum RU is too high or the minimum RU is too low. The `max_RU_tol` and `min_RU_tol` arguments set these thresholds.

It is **highly** recommended that this software be run on a high-performance platform. The compute-intensive processes are all parallelized. The software automatically detects the number of cores *actually available to the process* using `parallelly::availableCores()`, which respects the cgroup/Slurm/affinity limits imposed inside containers and on HPC schedulers (unlike `parallel::detectCores()`, which reports the host’s full physical core count and can lead to oversubscription). The user can change this to their preferred value via the `num_cores` argument to `process_input`.

On Unix/macOS, `num_cores = 20` is a good default, and the optimum scales roughly with the square root of the number of wells being fit (`cores ~ 1.7 * sqrt(wells)`, capped at the cores available): a small chip needs only a handful of cores, while a full 384-spot chip is best served by about 16–20. In benchmarks the total run time levels off around this “knee” (a full chip runs in ~337 s at 20 cores vs ~320 s at 40), after which only the curve-fitting stage keeps getting faster while the memory peak climbs steeply — so pushing past ~20 cores buys only a few percent. The backend is chosen automatically — forked workers (`parallel::mclapply`) on Unix/macOS, which share the large time-series data copy-on-write, and a PSOCK cluster on Windows. On Windows, prefer a moderate core count, since there the data must be serialized to each worker.

`create_pdf()` additionally accepts its own `num_cores` argument, defaulting to the value chosen in `process_input`. This is the most memory-intensive stage (it forks workers over many small plot objects), so on a memory-constrained machine you can pass it a lower core count than the rest of the pipeline — e.g. `create_pdf(..., num_cores = 8)` — to cap the memory peak with little effect on total run time. For a single full-chip run the whole pipeline peaks around 20 GB at 20 cores (~32 GB at 40), well within 64 GB.

When processing many chips, run **each analysis in a fresh R session** (e.g. a separate `Rscript` per chip) rather than looping many runs in one long-lived session. A long-lived session accumulates and fragments the R heap across runs, which both slows every stage (~1.5–1.8× at full chip) and inflates the memory peak dramatically — the same 384-well/40-core run that peaks ~32 GB in a fresh session reached ~109 GB inside a long multi-run session.

There are three default output files:

1. A `.pdf` file with each page corresponding to a spot on the chip. For each spot, there is a sensorgram with the baseline corrected data and the fitted RU curve for each concentration used to fit the curves/parameters, a residual plot, a response curve showing the selected concentrations and a table of kinetics parameters and their standard errors.
2. A `.pdf` file that tracks any errors encountered in fitting/plotting
3. A `.csv` file that outputs all fitted parameters and their standard errors

The software uses the data directory as the default output path, and uses the sample information filename along with a time/date stamp to name each output file. These can be changed by the user via arguments to `process_input`

### Output

As explained in the previous section, the standard pipeline outputs \(3\) files, a `.pdf` consisting of plots and tables for each spot on the chip:

### The Pipeline

```
# CRAN has strict limits on package sizes, and the titration files tend to be
#large. Therefore, we will need to download the titration data from the gitlab 
#repository. The following will download necessary files from gitlab onto
#your computer.

# By default, the files will go to the temp directory for this R session. 
# To change this, change the filename in destfile 
# Prepare destination path
destfile <- file.path(tempdir(), "titration_data.xlsx")

# Try downloading the file, handle errors gracefully
download_success <- tryCatch({
  download.file(
    "https://gitlab.oit.duke.edu/janice/htrspranalysis/-/raw/master/inst/extdata/titration_data.xlsx?ref_type=heads",
    destfile = destfile,
    mode = "wb"
  )
  TRUE
}, error = function(e) {
  message("Failed to download titration_data.xlsx: ", conditionMessage(e))
  FALSE
}, warning = function(w) {
  message("Warning during download: ", conditionMessage(w))
  FALSE
})

# Check if download was successful
if (!download_success || !file.exists(destfile)) {
  stop("Could not retrieve titration data. Please check your internet connection or the resource URL.")
}

# Locate sample sheet from installed package
sample_sheet_path <- system.file("extdata", "sample_sheet.xlsx", package = "htrSPRanalysis")

if (sample_sheet_path == "") {
  stop("Sample sheet not found in package 'htrSPRanalysis'. Is the package installed correctly?")
}

# Store path to downloaded data
data_file_path <- destfile

output_file_path <- tempdir()

# process the input
processed_input <- process_input(sample_sheet_path,
                    data_file_path,
                    num_cores = 2,
                    output_file_path = output_file_path)
```

### Obtain plots of raw data

```
plots_before_processing <- get_plots_before_baseline(processed_input)
```

Example plot:

```
plots_before_processing[[1]]
#> Warning: Removed 225 rows containing missing values or values outside the scale range
#> (`geom_point()`).
```

### Fit the data to the 1:1 Languir model

```
fits_list <- get_fits(processed_input)
```

### Plot the sensorgrams with fitted curves

```
plot_list <- get_fitted_plots(processed_input, fits_list)
```

Example plot:

```
plot_list[[1]]
```

### Create the response curve plots

These plots show the average response vs \(log\_{10}\) of the concentration of analyte. The color indicates the concentrations selected for fitting. These should be within the linear part of the curve.

```
rc_list <- get_rc_plots(processed_input)
```

Example plot

```
rc_list[[5]]
```

### Create a pdf with fitted sensorgrams, curve parameters, standard errors, residual plot and response curve.

```
pdf_pages <- create_pdf(processed_input, fits_list, rc_list, plot_list)
```

### Create a .csv file with all model parameters and standard errors.

```
parameter_table <- create_csv(processed_input, fits_list)
```

## Bulk shift example

### Pipeline

```
sample_sheet_path <- system.file("extdata", "bulkshift_example_sample_sheet.xlsx", package="htrSPRanalysis")
data_file_path <- system.file("extdata", "bulkshift_example_titration_data.xlsx", package="htrSPRanalysis")

# process the input

pdfpath <- tempdir()


bulkshift_example <- process_input(sample_sheet_path = sample_sheet_path,
                data_file_path = data_file_path,
                num_cores = 2, 
                output_file_path = pdfpath)
#> New names:
#> • `` -> `...1`
#> • `` -> `...2`
#> • `` -> `...3`
#> • `` -> `...4`
#> • `` -> `...5`

bulkshift_plots_before_processing <- get_plots_before_baseline(bulkshift_example)

bulkshift_fits <- get_fits(bulkshift_example)

bulkshift_plots <- get_fitted_plots(processed_input = bulkshift_example,
                                    fits_list = bulkshift_fits)

bulkshift_rc <- get_rc_plots(processed_input = bulkshift_example)
```

### Output

```
bulkshift_plots_before_processing[[1]]
#> Warning: Removed 114 rows containing missing values or values outside the scale range
#> (`geom_point()`).
```

```
bulkshift_plots[[1]]
#> Warning: Removed 1 row containing missing values or values outside the scale range
#> (`geom_point()`).
```

```
bulkshift_rc[[1]]
```

```
create_pdf(processed_input = bulkshift_example,
           fits_list = bulkshift_fits,
           rc_list = bulkshift_rc,
           plot_list = bulkshift_plots)
#> Warning: Removed 1 row containing missing values or values outside the scale range
#> (`geom_point()`).

create_csv(processed_input = bulkshift_example,
           fits_list = bulkshift_fits)
#> # A tibble: 1 × 42
#>   Ligand Analyte Block Row   Column   ROI Rmax1 Rmax2 Rmax3 Rmax4 Rmax5 Rmax_se1
#>   <chr>  <chr>   <dbl> <chr>  <int> <dbl> <dbl> <dbl> <dbl> <dbl> <dbl>    <dbl>
#> 1 Antib… Antige…     1 A          1     1  59.9  72.0  83.5  87.8  91.4     2.78
#> # ℹ 30 more variables: Rmax_se2 <dbl>, Rmax_se3 <dbl>, Rmax_se4 <dbl>,
#> #   Rmax_se5 <dbl>, ka <dbl>, ka_se <dbl>, kd <dbl>, kd_se <dbl>, KD <dbl>,
#> #   KD_se <dbl>, Bulkshift1 <dbl>, Bulkshift2 <dbl>, Bulkshift3 <dbl>,
#> #   Bulkshift4 <dbl>, Bulkshift5 <dbl>, Bulkshift_se1 <dbl>,
#> #   Bulkshift_se2 <dbl>, Bulkshift_se3 <dbl>, Bulkshift_se4 <dbl>,
#> #   Bulkshift_se5 <dbl>, R01 <dbl>, R02 <dbl>, R03 <dbl>, R04 <dbl>, R05 <dbl>,
#> #   R0_se1 <dbl>, R0_se2 <dbl>, R0_se3 <dbl>, R0_se4 <dbl>, R0_se5 <dbl>
```

## Upward drift example

Upward drift occurs when the signal rises during the dissociation phase rather than decaying monotonically. This can indicate non-specific binding, surface instability, or instrument artifacts. The software automatically detects upward drift and flags affected spots.

### Pipeline

```
sample_sheet_path <- system.file("extdata", "updrift_test_sample_sheet.xlsx", package = "htrSPRanalysis")
data_file_path <- system.file("extdata", "updrift_test_titration_data.xlsx", package = "htrSPRanalysis")

pdfpath <- tempdir()

updrift_example <- process_input(sample_sheet_path = sample_sheet_path,
                data_file_path = data_file_path,
                num_cores = 2,
                output_file_path = pdfpath)

updrift_plots_before_processing <- get_plots_before_baseline(updrift_example)

updrift_fits <- get_fits(updrift_example)

updrift_plots <- get_fitted_plots(processed_input = updrift_example,
                                  fits_list = updrift_fits)

updrift_rc <- get_rc_plots(processed_input = updrift_example)
```

### Output

The example dataset contains 5 spots with varying drift rates (0, 0, 0.03, 0.12, and 0.07 RU/s). Spots 4 and 5, which have strong or moderate upward drift, are flagged automatically.

```
updrift_plots_before_processing[[4]]
```

```
updrift_plots[[4]]
#> Warning: Removed 4 rows containing missing values or values outside the scale range
#> (`geom_point()`).
```

```
updrift_rc[[4]]
```

```
create_pdf(processed_input = updrift_example,
           fits_list = updrift_fits,
           rc_list = updrift_rc,
           plot_list = updrift_plots)

create_csv(processed_input = updrift_example,
           fits_list = updrift_fits)
#> # A tibble: 5 × 42
#>   Ligand Analyte Block Row   Column   ROI Rmax1 Rmax2 Rmax3 Rmax4 Rmax5 Rmax_se1
#>   <chr>  <chr>   <dbl> <chr>  <int> <dbl> <dbl> <dbl> <dbl> <dbl> <dbl>    <dbl>
#> 1 Antib… Antige…     1 A          1     1  200.  200.  200.  200.    NA    0.262
#> 2 Antib… Antige…     2 A          1     2  150.  150.  150.  150.    NA    0.102
#> 3 Antib… Antige…     3 A          1     3  172.  174.  176.  177.    NA    0.443
#> 4 Antib… Antige…     4 A          1     4  147.  150.  154.  156.    NA    0.277
#> 5 Antib… Antige…     1 A          2     5  208.  207.  210.  213.    NA    0.745
#> # ℹ 30 more variables: Rmax_se2 <dbl>, Rmax_se3 <dbl>, Rmax_se4 <dbl>,
#> #   Rmax_se5 <dbl>, ka <dbl>, ka_se <dbl>, kd <dbl>, kd_se <dbl>, KD <dbl>,
#> #   KD_se <dbl>, Bulkshift1 <dbl>, Bulkshift2 <dbl>, Bulkshift3 <dbl>,
#> #   Bulkshift4 <dbl>, Bulkshift5 <dbl>, Bulkshift_se1 <dbl>,
#> #   Bulkshift_se2 <dbl>, Bulkshift_se3 <dbl>, Bulkshift_se4 <dbl>,
#> #   Bulkshift_se5 <dbl>, R01 <dbl>, R02 <dbl>, R03 <dbl>, R04 <dbl>, R05 <dbl>,
#> #   R0_se1 <dbl>, R0_se2 <dbl>, R0_se3 <dbl>, R0_se4 <dbl>, R0_se5 <dbl>
```
